# Light in heart, forge ahead—Professor Rui Wang’s adventures in perovskite solar cell frontiers

**DOI:** 10.1038/s41377-025-01863-5

**Published:** 2025-04-28

**Authors:** Ji Wang

**Affiliations:** Westlake University, No. 600 Dunyu Road, Xihu District, Hangzhou, Zhejiang 310030 China

**Keywords:** Optics and photonics, Optical materials and structures

## Abstract

As depicted in ancient Greek mythology, Prometheus couldn’t bear the sight of humanity struggling in the darkness, crafted a long reed, and took the risk of approaching the sun to steal fire. He fearlessly brought this light to the world, defying what Zeus had ordered the gods not to do. His brave act ushered in the dawn of civilization for mankind. In this issue of “Light People”, Professor Rui Wang is invited to share stories about his adventures in improving perovskite solar cells for the full utilization of sunlight in daily lives, much like Prometheus bringing the gift of light to humanity. Perovskite solar cells hold great potential for both civilian applications and commercial purposes, such as rooftop solar panels, solar chargers, and solar-powered vehicles.

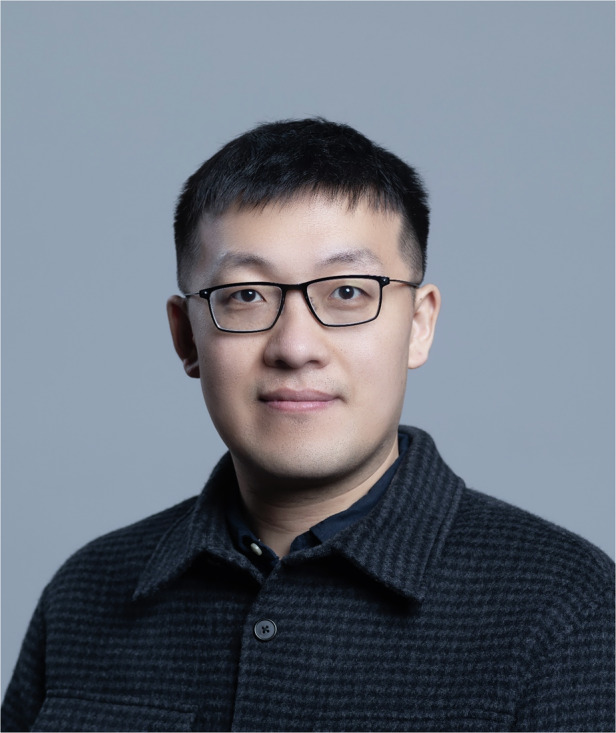



**Short Bio:**Rui Wang, an assistant professor and independent principal investigator at the School of Engineering, Westlake University, was born in 1993 in Jinzhou, Liaoning Province. In 2015, he obtained a Bachelor of Engineering degree from Jilin University, followed by a Master’s degree from the University of California, Berkeley, in 2016. From September 2016 to December 2019, he studied at the University of California, Los Angeles(UCLA), under the supervision of Professor Yang Yang, and was awarded a doctorate. Subsequently, he continued his postdoctoral research at UCLA. In April 2021, he joined the School of Engineering at Westlake University, on a full-time basis. He has been selected for the Forbes 30 Under 30-China and Asia, the MIT Technology Review 35 Innovators Under 35, the DAMO Academy’s Orange Award, and ESI Highly Cited Researchers (Cross-Field).

**Q1: When you were a little child, what were your dreams? Who inspired you to become a young scientist?**

**A1:** My parents worked for a solar cell company for almost 20 years. Growing up in a family deeply involved in industries of renewable energy, I have been immersed in exploring the solar power from a young age. Once, while accompanying my parents on a business trip to the northwest region, I witnessed the solar cells produced by their company being installed in one household after another. The families were filled with curiosity about the unknown and a longing for a more convenient life. Their excitement and gratitude were palpable as they embraced this new technology. This experience left a profound impression on me. I realized that technology is not just a collection of abstract concepts and theories; it is a powerful force that can transform lives and shape the future. It was during this trip that I planted the seed of becoming a professional dedicated to promoting the development of the solar energy industry.

The idea of becoming a scientist is like a beacon, illuminating the path of my quest for knowledge and guiding me on this journey filled with unknowns and challenges, making my steps more resolute and far-reaching. To realize this dream, I pursued advanced studies at Jilin University, the University of California, Berkeley, and the University of California at Los Angeles. Each of these educational experiences served as a solid foundation, supporting my ascent in the scientific world. After completing my doctoral studies, I joined Westlake University, where I have dedicated myself entirely to research in renewable energy technologies, particularly in the field of novel solar cells. My work has been a witness to significant progress in this area.

I am deeply grateful for the life and educational experiences that have shaped my career path. These experiences have not only equipped me with the necessary skills and knowledge but also instilled in me a sense of purpose and determination. Science is not merely a process of exploring the arcane and off-the-beaten-path things but a means of serving society and shaping human life as well. Becoming a scientist is a beautiful way to realize my self-worth. Each and every successful or unsuccessful experiment is a step toward the truth; each and every published paper is a tribute to the work of predecessors and a guide for future explorers. My goal is to drive the advancement of solar technology, enhancing its efficiency, stability, and accessibility for people around the world.

**Q2: How did your academic study at Jilin University, the University of California, Berkeley, and the University of California, Los Angeles, where you obtained your bachelor’s, master’s, and doctoral degrees, respectively, influence your academic and professional development?**

**A2:** At Jilin University, where I earned my bachelor’s degree, I was introduced to the foundational knowledge and rigorous academic discipline that set the stage for my future studies. The strong emphasis on theoretical understanding and practical skills provided me with a solid foundation in science, essential for any researcher or academic. Moreover, the environment fostered critical thinking and encouraged curiosity, which are invaluable traits in scientific research. The comprehensive curriculum and dedicated faculty at Jilin University laid the groundwork for my lifelong pursuit of knowledge and innovation.

Moving to the University of California, Berkeley for my master’s degree marked a significant transition in my academic life. Berkeley is renowned for its cutting-edge research and diverse intellectual community. This exposure broadened my perspective and deepened my understanding of my field of study. During this period, the opportunities to engage with leading experts and participate in advanced research projects greatly enhanced my technical skills and research methodologies. It also instilled in me a passion for pursuing innovative solutions to complex problems. The collaborative and dynamic atmosphere at Berkeley challenged me to think critically and creatively, preparing me for the rigors of advanced research.

Pursuing my doctoral degree at the University of California, Los Angeles (UCLA) further distilled my research focus and solidified my professional identity. UCLA’s commitment to interdisciplinary research and collaboration opened doors to new areas of inquiry and methodologies. My dissertation research empowered me to conduct an in-depth exploration of particular questions germane to my field, yielding original knowledge and potentially far-reaching findings. The mentorship bestowed upon me by the erudite faculty members at UCLA played a pivotal role in cultivating my ability to foster independent thinking, manage intricate projects, and disseminate profound scientific ideas. The interdisciplinary approach at UCLA not only enriched my research but also prepared me to address multifaceted challenges in the scientific community.

My journey through Jilin University, Berkeley, and UCLA has not only endowed me with the essential knowledge and skills for my profession but has also fostered a deep sense of duty to contribute to the field and create favorable social impacts through my research.

**Figure:**
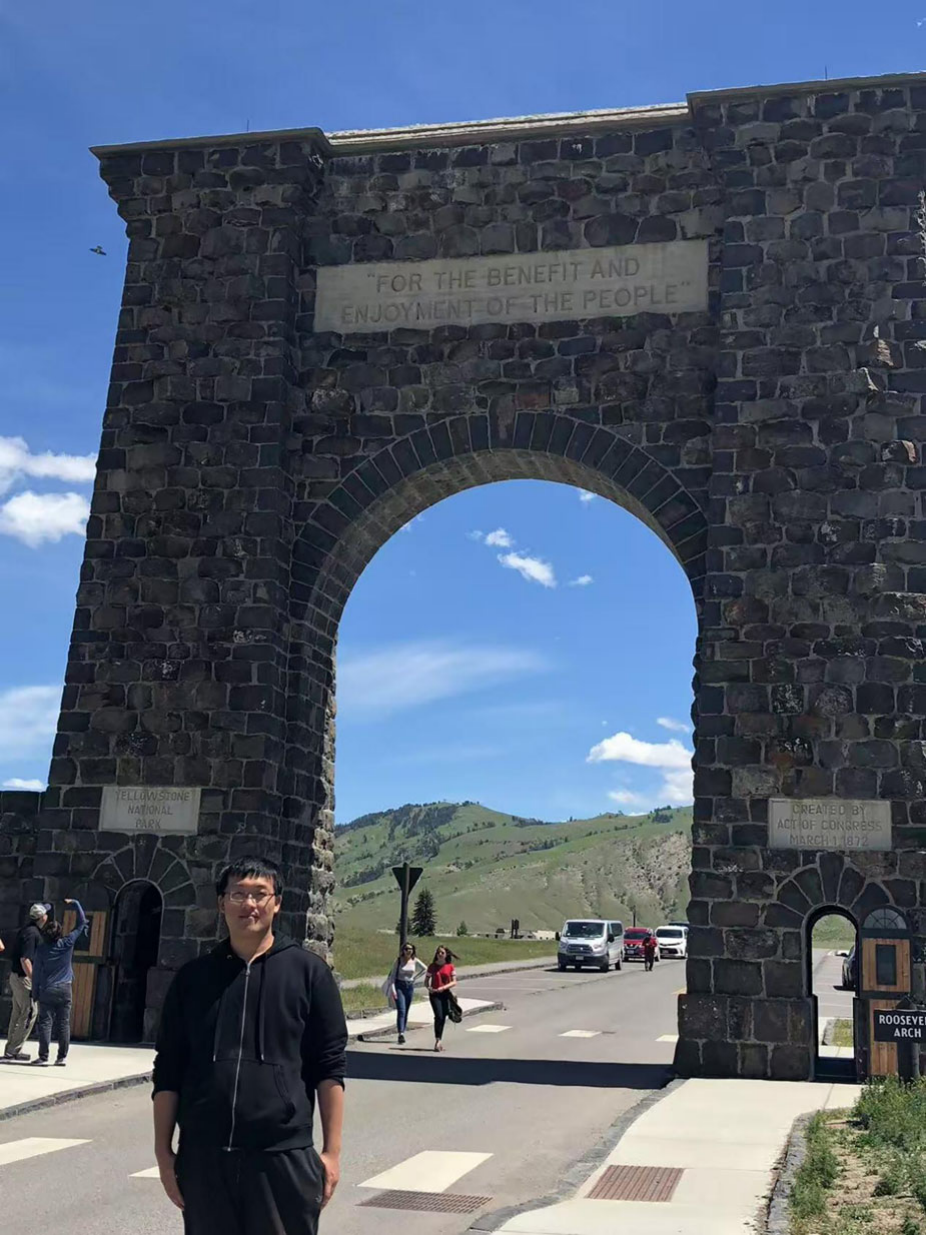
Rui Wang’s Post-Newport-Certification Visit to Yellowstone Park

**Q3: From your perspective, how do the different approaches to nurturing scientific creativity in science education between China and the United States shape students’ ability to solve complex real-world problems in the era of rapid technological transformation?**

**A3:** In China, the science education system emphasizes a strong foundation in theoretical knowledge and systematic training. This approach cultivates students’ analytical skills and ability to work within structured frameworks. For instance, during my time at Jilin University, I witnessed a focus on mastering the basics before diving into specialized research. This foundation is crucial, enabling students to understand complex scientific concepts and apply them in practical settings. On the other hand, the American science education system tends to encourage more open-ended exploration and creativity. American schools often provide students with ample opportunities to engage in hands-on projects, experiment with new ideas, and think outside the box. This approach is vividly reflected in how American educators encourage students to pose their hypotheses, conduct experiments, and analyze data independently.

In my research journey, I’ve found that combining the strengths of both systems can yield remarkable results. My work on perovskite solar cells has benefited greatly from the rigorous theoretical training I received in China and the creative, experimental approach to research that I encountered in the United States. This blend has allowed me to innovate within a solid scientific framework, pushing the boundaries of what’s possible in solar energy technology.

In conclusion, both Chinese and American science education systems have their unique strengths in fostering scientific creativity. Integrating the exemplary practices from both Eastern and Western educational paradigms will nurture a cohort of students, who are not only well-versed in extant knowledge, but also capable of generating novel ideas and adeptly solving complex real-world problems in the era of rapid technological transformation.

**Q4: During your time of learning and researching under Professor Yang Yang, who is renowned for inventing inverted organic solar cells and transparent organic photovoltaic devices, are there any particularly engaging stories or unexpected moments that stand out in your memory?**

**A4:** In 2018, when my research was just beginning, the power conversion efficiency of the perovskite solar cells was around 18%. I enjoy drinking coffee, and at that time, I often engaged in discussions with my team members over a cup of coffee.

On one occasion, a sudden spark of inspiration jolted me, while we leisurely sipping our coffee: coffee stabilizes human emotions, so could it potentially stabilize the “mood” of perovskite materials and enhance their stability? After consulting relevant literature, we discovered that caffeine contains two functional groups, which resemble those found in previous studies to control perovskite crystallization and promote material growth. Consequently, we initially hypothesized that caffeine might contribute to improving the performance of perovskite solar cells. Having validated the effectiveness of caffeine, we delved deeper into its specific impact mechanism on the performance of perovskite solar cells and found that caffeine’s rigid structure and higher boiling point could compensate for the deficiencies of certain small molecules in perovskite solar cells, thereby enhancing the cells’ efficiency and stability. It was a very intriguing idea, and after conducting experiments, we found that the efficiency of the solar cells increased to 20%.

**Figure:**
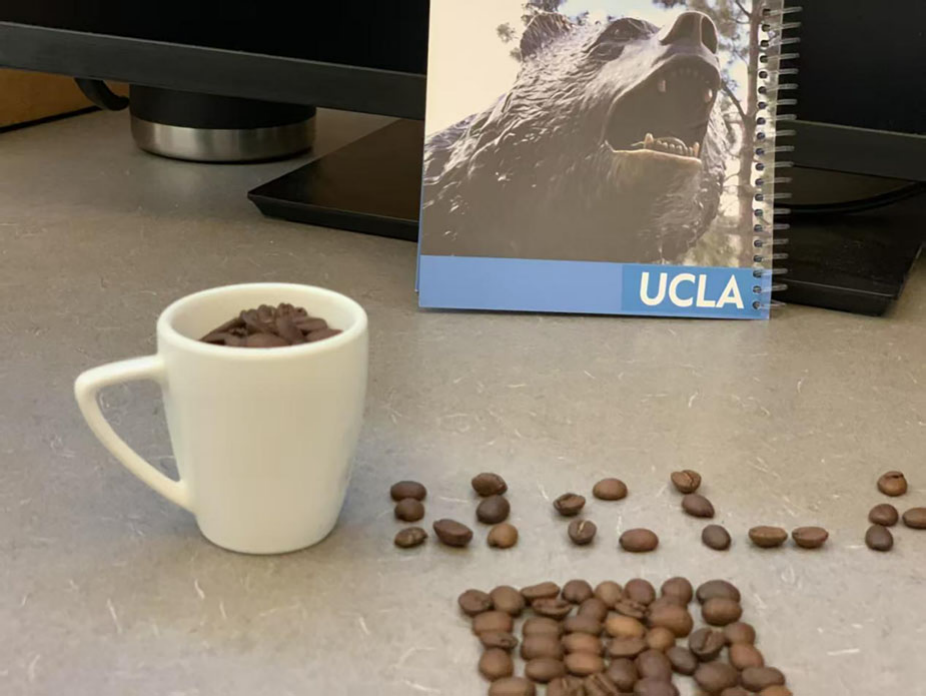
Professor Rui Wang Drawing Inspiration from Caffeine to Improve Perovskite Solar Cells’ Stability

**Q5: Your research group published a total of 6 papers in high-impact journals —*Nature*** **and** ***Science***^1–6^ **with the focus on various aspects of perovskite photovoltaic devices, including their fabrication, characterization, stability, defect mechanisms, exploration of new organic-inorganic hybrid photovoltaic materials, and other solar cell studies***.* **Could you highlight some of the recent breakthroughs or advancements in each of these research directions?**

**A5:** Numerous reports attest that our research team and collaborators are currently deeply engaged in the investigation of inverted perovskite solar cell devices, with a pioneering focus on Py3, a new hole-selective material meticulously designed with a large conjugated pyrene ring as its core structure. This groundbreaking work has recently been published in the prestigious journal *Nature*.

Even though our group is not the first to explore the design of such molecules, it is of great significance that we carry out in-depth and far-reaching research in this area. The literature reported a variety of hole-selective molecules, which are predominantly designed with the employment of carbazolyl or trianiline groups. These molecules typically incorporate an alkyl phosphonic acid functional group at their termini. This group serves as the anchor point for direct bonding with the transparent electrode, thereby enabling the formation of a hole-selective contact structure.

However, a critical limitation of these previously studied molecules is their inherent instability, stemming from the presence of heteroatoms within their parent nuclei. These heteroatoms serve as potential sites for material degradation, posing significant challenges to the long-term stability and performance of perovskite solar cells. In our research, we have made a significant advancement by innovatively introducing a heteroatom-free pyrene ring as the parent core of the hole-selective material. Not only does this strategic choice enhance the stability of the device’s functional layer, but it also addresses the instability issues associated with previous hole-selective materials. Furthermore, our work represents a paradigm shift in the field by revealing that the hole-selective contact structure is not a monolithic single-molecular layer, as previously postulated. Instead, it is a complex multi-layer structure formed through the accumulation of molecules. This groundbreaking discovery underscores the crucial role of *π*-*π* stacking of molecular parent nuclei in carrier transport within this layer. The dependence on such strongly conjugated stacking systems further emphasizes their indispensable importance in ensuring efficient and stable charge extraction and transport within perovskite solar cells.

Our findings not only contribute to a deeper understanding of the hole-selective contact structure in perovskite solar cells but also pave the way for the development of more stable and efficient inverted perovskite solar cell devices.

**Figure:**
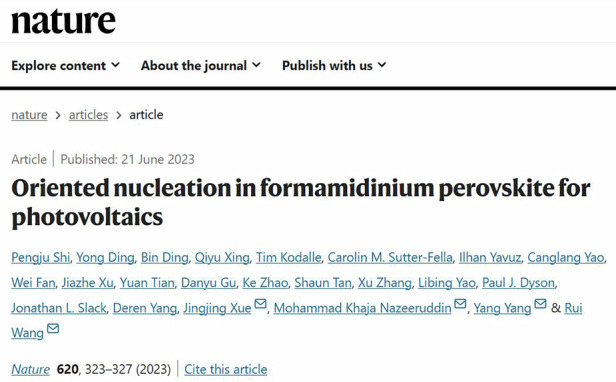
Research Papers Authored by Professor Rui Wang and His Team^3^

**Q6: What challenges and chances have you encountered during your research on perovskite photovoltaic devices?**

**A6:** Currently, perovskite solar cells still face several technical challenges awaiting resolution. The most pressing challenge is the light-absorbing layer’s thermal instability, as it undergoes hydrolysis and decomposition at elevated temperatures, leading to performance degradation. The reported longest operational lifespan of perovskite cells typically reaches merely a few thousand hours, far shorter than that of crystalline silicon cells. Another example is that the perovskite cells produced in laboratories are typically small, which does not meet the market demand for larger solar cells. Moreover, perovskite materials feature a short crystallization time, with the production process window lasting merely a few seconds, which presents substantial challenges to the manufacturing process.

However, perovskite solar cells exhibit a multitude of remarkable advantages, such as high conversion efficiency, low production cost, short manufacturing cycles, excellent low-light performance, and lightweight and portable design. With the development of tandem cells, the conversion efficiency of perovskite cells is anticipated to be further enhanced. We have already obtained a steady-state PCE of 23.28% (certified 22.79%) for flexible monolithic perovskite/Cu (In, Ga) Se_2_ tandem cells^7^. Moreover, numerous national-level policies have been issued to actively promote the development of perovskite solar cells, including “The 14th Five-Year Plan for Scientific and Technological Innovation in the Energy Sector”, “The Implementation Plan for Carbon Peak and Carbon Neutrality Supported by Science and Technology(2022-2030) ”, and “The Guidance on Promoting the Development of Energy Electronics Industry(Draft for Solicitation of Opinions)”.

Therefore, my team and I continue to leverage our strengths and concentrate our efforts on improving the stability and extending the lifespan of the materials. We also conduct in-depth cooperation and exchanges with other domestic and international scientific research institutions and enterprises, jointly promoting the progress and application of perovskite solar cell technology.

**Q7: At the age of 28, you became an independent principal investigator and assistant professor at Westlake University. What are your expectations for future optical innovation in China?**

**A7:** Looking ahead, I am incredibly excited about the future of optical innovation in China. Our country has already made significant progress in this field, with many groundbreaking discoveries and technological advancements. However, I believe that we are just at the beginning of a new era of optical innovation. With the rapid development of technology and the increasing demand for advanced optical solutions, I am highly anticipative of witnessing a burgeoning stream of innovative ideas and technologies originating from China. Here are our expectations:

- With the growing investment in scientific research, we can delve deeper into the fundamental principles of light-matter interactions. This will provide a solid theoretical foundation for the development of new technologies, particularly in such areas as nonlinear optics and quantum optics.
- The integration of optics with other disciplines, including materials science, life sciences, and computer science is set to drive a multitude of innovative applications and technological breakthroughs. Examples can be seen in the advancement of bioimaging techniques, such as super-resolution microscopy, and in quantum communication protocols, such as quantum key distribution.
- China’s robust manufacturing capabilities and well-established supply chain system facilitate the transition of optical technologies from the laboratory to the market. We anticipate the industrialization of more core technologies with independent intellectual property rights, particularly in the development of high-efficiency photovoltaic cells and advanced optical sensors.
- Strengthening domestic and international academic exchanges and collaborations will attract and cultivate a cadre of high-level researchers. This will contribute to the advancement of optical technology both in China and globally, and promote the growth of multidisciplinary research centers and innovation hubs.
- The government’s increasing support for technological innovation, particularly in high-tech fields, ensures ample funding and a favorable policy environment. This will significantly accelerate the research and development process in optical technologies, including photonics, nanophotonics, and optoelectronics.

By fostering a culture of open collaboration and knowledge sharing, we could create an environment that nurtures creativity and encourages the development of new ideas. As a researcher and educator, I am committed to contributing to this exciting journey of optical innovation in China. I look forward to working with my colleagues and students to explore new frontiers in optics and to make meaningful contributions to the scientific community and society at large.

**Figure:**
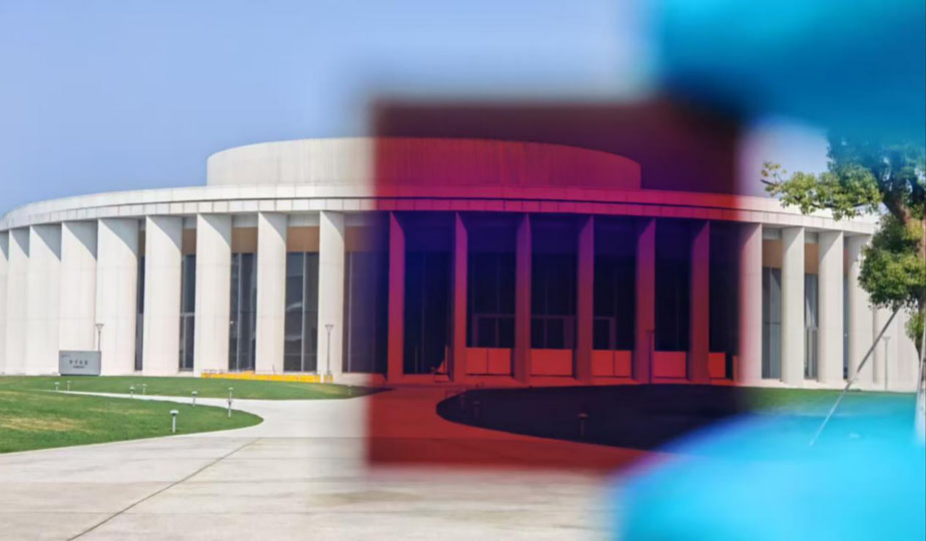
Semitransparent Perovskite Solar Cells at Westlake University

**Q8: Having been selected for the Forbes 30 Under 30-China and Asia and the MIT Technology Review 35 Innovators Under 35, along with other accolades, what insights or words of wisdom would you like to share with today’s youths, who seem to be prone to a “lying-flat” attitude in this highly competitive age?**

**A8:** I am truly honored and grateful to receive these accolades and the encouragement they bring. However, these accolades are not the end goals; they are merely milestones in a much longer journey of my exploration and discovery. In a world that often seems overwhelming and competitive, it’s easy to feel discouraged or adopt a “lying flat” mindset, where one passively gives up on their ambitions with minimal resistance. While there’s nothing inherently wrong with smelling the roses and taking time to recharge, it’s crucial to recognize the importance of setting goals and pushing oneself to achieve them.

Following the receipt of these honors, I have come to deeply realize that continuous effort and unwavering dedication are crucial, whether in scientific research or personal growth. In this rapidly changing era, facing various challenges and pressures, some young people may feel exhausted and even inclined to “lie flat”. However, I want to emphasize that no effort in life is in vain; every step eventually contributes to the path to success.

Firstly, I believe that maintaining a sense of curiosity and a thirst for knowledge is essential. Curiosity drives us to explore unknown territories, while a thirst for knowledge motivates us to invest time and energy in deepening our understanding and mastering new information.

Secondly, the courage to accept challenges and the resilience to face failure are equally indispensable. In the realm of scientific research, encountering difficulties and setbacks is inevitable. However, if we can learn from these experiences, stand up with courage, and keep moving forward, there is nothing that cannot be overcome. Each attempt, even if it fails, is a valuable accumulation of experience.

Furthermore, I want to highlight the importance of teamwork. In modern scientific research, few achievements are accomplished by a single individual. The strength of a team enables us to go further. Mastering the art of synergizing with others, sharing resources, and supporting each other does more than merely facilitate the success of projects; it fosters lasting friendships in the process.

All in all, to the young people who might be feeling overwhelmed or disillusioned by the pressures of modern society, I would like to say: find your passion, set realistic goals, never be content with mediocrity, and never give up on your dreams. For me, researching solar cells is a journey toward light, and my goal is to achieve a groundbreaking discovery from 0 to 1.

**Figure:**
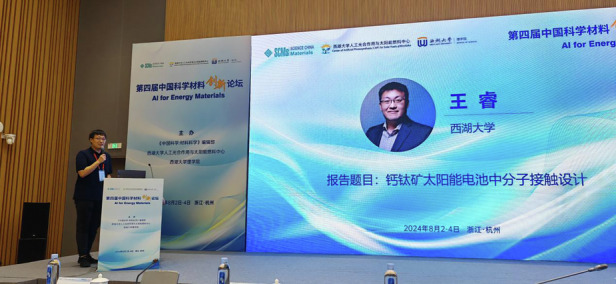
Professor Rui Wang’s Presentation at the Materials Innovation Forum

**Q9: Being a scientist is quite demanding, so how do you strike a balance between your professional career and private life?**

**A9:** Although scientific research demands considerable time and energy, I believe it is crucial to find a healthy balance between one’s career and personal life to maintain long-term creativity and contentment. Striking this balance isn’t merely about ensuring personal well-being; it also significantly bolsters professional performance and cultivates a sustainable career progression.

First of all, I practice strict time management, carefully planning work and leisure time. This structured approach helps me maintain a high level of productivity, while also allowing me ample time to enjoy my personal life. Effective time management is essential in a field as demanding as scientific research, where deadlines and experiments can be unpredictable. By setting clear boundaries and sticking to a schedule, I ensure that I can meet my professional obligations without neglecting my personal needs.

Secondly, I prioritize communication with my family and friends. Maintaining strong relationships is vital for emotional support and overall well-being. During the holidays, I choose to return home to spend some quality time with my parents. They are my most important supporters, and their understanding and support enable me to focus more on scientific research. Regularly reconnecting with loved ones keeps me grounded and highly motivated. It endows me with a sense of stability and belonging, both of which are vital in a high-pressure environment.

Thirdly, given the paramountcy of health, I regulate certain lifestyle habits like regular exercise, a balanced diet, and sufficient sleep to ensure a healthy body. Physical well-being directly impacts mental clarity and resilience, both of which are essential qualities for a scientist. By taking care of my body, I enhance my ability to tackle complex problems and maintain a consistent level of performance.

Last but not least, an open-and-flexible mindset is what I always hold fast to. As my career progresses and my personal life changes, I need to continually adjust my balance point. Flexibility allows me to adapt to new challenges and chances, ensuring that I remain both professionally and personally fulfilled. Whether taking on a new project, acquiring a new skill, or simply trying a new hobby, I embrace change as a chance to grow.

By integrating these tips into my daily life, I strive to create a harmonious balance that supports both my professional aspirations and personal well-being.

**Q10: What is your inspiring motto that keeps you going in your optics research? In what ways do you inspire your team to aspire to excellence?**

**A10:** “Light in heart, forge ahead” is not merely my motto, but also my earnest expectation for young researchers.

“Light in heart” for me, signifies the ideals and beliefs in scientific research. It stems from curiosity and exploration of the unknown world, representing an unwavering pursuit of scientific truth. Thomas Edison experienced thousands of failures when inventing the light bulb, yet he never gave up and eventually brought light to the world. On the path of scientific research, we often encounter setbacks and failures, but it is this inner light that illuminates us forward, enabling us to be undaunted by hardships and bold in embracing challenges. It instills in us the unwavering belief that every endeavor and effort is a solid step towards success.

“Forge ahead” serves as a reminder of our actions. In the journey of scientific research, there are absolutely no smooth paths nor shortcuts; instead, steep mountains to climb, one after another. We should clarify our research goals and directions, far from the hustle and bustle of the outside world; we should hone our will and abilities constantly, just as a whetstone sharpens a blade; we should explore, endeavor, experiment, and execute. This process makes us more sharper-witted and resolute on the arduous journey of scientific research.

I inspire my PhD students to build up their confidence to take on research challenges independently and explore questions as thoroughly and deeply as they can. Through efficient feedback and personalized mentorship, I lead students to overcome obstacles, refine their approaches, and eventually advance their research progress.

“Light in heart, forge ahead.” May we all carry the light within our hearts, bravely walk on the path of scientific research, continuously surpass ourselves, and contribute our strength to the advancement of human technology.

**Figure:**
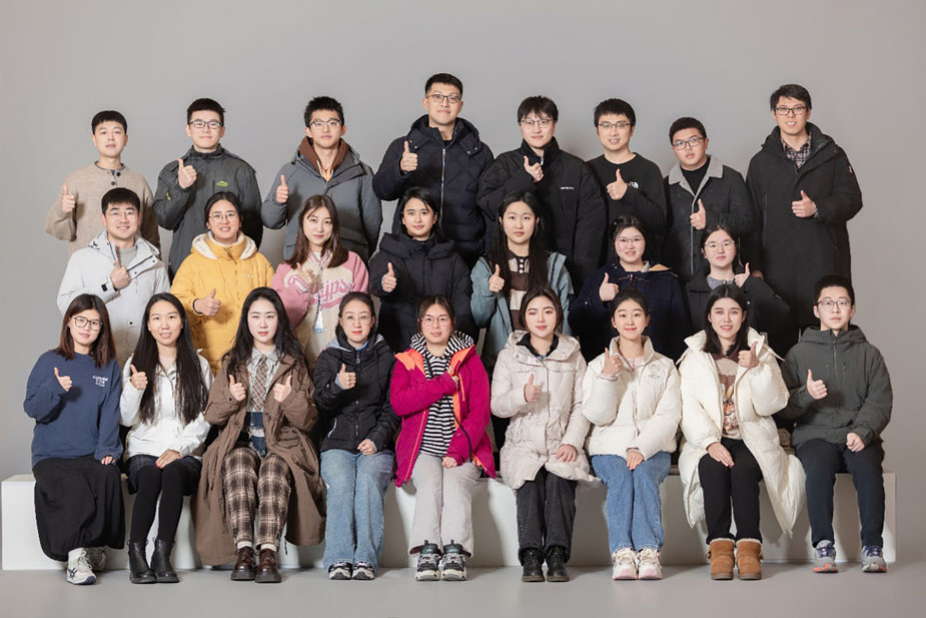
Professor Rui Wang and His Team at Westlake University
